# Impact of *LMP7* (rs2071543) gene polymorphism in increasing cancer risk: evidence from a meta-analysis and trial sequential analysis

**DOI:** 10.18632/oncotarget.23547

**Published:** 2017-12-21

**Authors:** Raju K. Mandal, Sajad A. Dar, Arshad Jawed, Mohd Wahid, Mohtashim Lohani, Aditya K. Panda, Bhartendu N. Mishra, Naseem Akhter, Mohammed Y. Areeshi, Shafiul Haque

**Affiliations:** ^1^ Research and Scientific Studies Unit, College of Nursing and Allied Health Sciences, Jazan University, Jazan 45142, Saudi Arabia; ^2^ The University College of Medical Sciences and GTB Hospital University of Delhi, Delhi 110095, India; ^3^ Department of Biosciences, Faculty of Natural Sciences, Jamia Millia Islamia A Central University, New Delhi 110025, India; ^4^ Centre for Life Sciences, Central University of Jharkhand, Ranchi, Jharkhand 835205, India; ^5^ Department of Biotechnology, Institute of Engineering and Technology, Lucknow, Uttar Pradesh 226021, India; ^6^ Department of Laboratory Medicine, Faculty of Applied Medical Sciences, Albaha University, Albaha 65431, Saudi Arabia

**Keywords:** meta-analysis, LMP7, cancer susceptibility, polymorphism, trial sequential analysis

## Abstract

Genetic variant *LMP7* (low molecular weight polypeptide 7) –145 C > A may influence the function of immune surveillance of an individual and lead to cancer development. Various studies have investigated the relevance of *LMP7* –145 C > A gene polymorphism with cancer risk; but, their results are conflicting and inconsistent. To obtain a comprehensive conclusion, a meta-analysis was performed by including eight eligible published studies retrieved from PubMed (Medline), EMBASE and Google Scholar web search until December 2016. Individuals with AA genotype (AA vs CC: *p* = 0.001; OR = 2.602, 95% CI = 1.780 to 3.803) of LMP7 -145 C > A were found to have 2 folds higher risk of cancer than those with CC genotype. The recessive genetic model (AA vs AC + CC) also indicated that individuals with AA genotype have 2 folds higher cancer risk than AC and CC genotypes (*p* = 0.001; OR = 2.216, 95% CI = 1.525 to 3.221). Also, significant increased cancer risk was observed in Asians but not in Caucasians. No publication bias was observed during the analysis. Trial sequential analysis also strengthened our current findings. These results suggest that genetic variant LMP7–145 C > A has significant role in increasing cancer risk in overall and Asian population, and could be useful as a prognostic marker for early cancer predisposition.

## INTRODUCTION

Cancer is one of the ever dreadful disease, and a leading cause of death, and a severe public health problem worldwide [1]. Various projection based studies reported that the worldwide burden of cancer will rise more rapidly with rising population, aging and changes in the lifestyle [2]. It is predicted that there will be more than 20 million new cancer cases globally by the year 2025 [3]. The increasing incidence and mortality rate due to cancer during the last two decades have posed a big challenge to the clinicians and scientists. Also, the precise mechanism of carcinogenesis has not been fully deciphered yet. In the recent years, the growing number of studies reported that the initiation of cancer is a complex process that includes the results of genetic susceptibility and various environmental factors [4]. Therefore, the identification of genetic risk factors that contribute to the substantial burden of the disease in general population is warranted for the development of broad therapeutic strategies for cancer prevention.

Recent genomewide association studies (GWAS), and other case-control studies have revealed that single-nucleotide polymorphisms (SNPs) are the most common forms of human genetic variations, have important role in defining susceptibility to cancer [5, 6]. This clearly suggests the SNPs can be used as a promising biomarker for the evaluation of individual genetic background for cancer prognosis, and signifies an interesting field of cancer research.

Major histocompatibility complex (MHC) is a set of cell surface glycoproteins that bind with the intracellularly processed peptides from pathogens, and present them on the surface of tumor cells to cytotoxic T lymphocytes (CTL). Therefore, MHC plays a key role in the initiation of CTL linked inflammation and anticancer immune response [7]. MHC restricted immune response has been tested to eliminate cancer cells, and the expression of MHC antigens may be important for the host immune response against cancer [8].

The low molecular weight polypeptide 7 (*LMP7*, also called as *PSMB8*) gene is located in the class II region of the MHC locus on chromosome 6 [9]. This gene encodes peptide forming the large components of the proteasome complex engaged in the degradation of cytosolic proteins and the generation of antigenic peptides [10, 11]. The components of the proteasome are thought to participate in the proteolytic digestion of cancer-relevant antigen peptides derived from endogenous or exogenous antigenic proteins, and play central role in homoeostasis of cellular proteins and regulation of cellular processes that are important in cancer initiation and progression [12].

Single-nucleotide polymorphism (SNPs) is one of the most common forms of genetic alterations. Several studies from TCGA and COSMIC were perused to analyze the association between the LMP7 gene mutations and risk of developing cancer including human breast and colorectal cancers [13], human glioblastoma multiforme [14], pancreatic cancer [15], melanoma [16], and human colon and rectum cancer [17]. Various polymorphic residues have also been identified in *LMP7* gene [18]. Previous studies have reported that *LMP7–*145 (Gln > Lys, C > A, rs2071543) gene polymorphism result in functional alternation, and ultimately deteriorate the capacity of antigen processing [19]. Thus, an abnormal expression of low molecular peptide (LMP) subunits attributes to many disease phenotypes and malignant tumors.

In view of the crucial restrictive role of *LMP7* gene in antigen processing and presentation, this gene is a promising candidate for carcinogenesis susceptibility. In the last few years, a number of case-control studies have investigated the impact of this gene polymorphism on cancer risk in various populations; but the reported results varied across studies and remain inconclusive [20–27]. Inconsistency in the findings of previous studies could be possibly attributed to small sample size, and low statistical power. Lately, Burton et al. (2009) reported that large sample size is always good, and necessary to study the genetic associations with complex diseases [28]. Therefore, in this study, a meta-analysis was performed by pooling all the eligible published studies to determine the more precise association and understanding the possible role of *LMP7–*145 (C > A) gene polymorphism as genetic marker for cancer progression. Further, the quality of the included studies was checked by performing Newcastle Ottawa Scale (NOS) analysis. Also, type-I statistical errors, i.e., publication bias and random errors occurred due to sparse data were minimized by performing Trial Sequential Analysis (TSA) for quantifying the statistical reliability of the data included in the cumulative meta-analysis with the threshold of statistical significance. Overall, a meta-analysis is a powerful statistical tool for analyzing cumulative data from different studies, in which the individual sample sizes are small and the statistical power is low, and it provides precise and robust conclusion [29].

## RESULTS

### Literature search and meta-analysis databases

**Figure 1:**
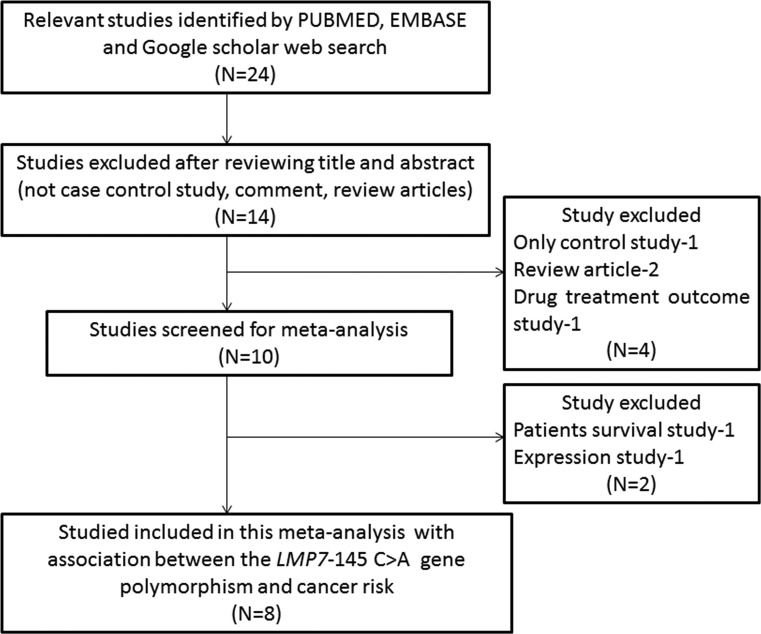
PRISMA (preferred reporting items for systematic reviews and meta-analyses) flow-diagram outlining the identification and selection of studies for inclusion/exclusion of the relevant studies in this meta-analysis

The confounding conclusions from previous studies regarding the role of *LMP7–*145 (C > A) gene polymorphism as genetic marker for cancer susceptibility and progression [20–27] impelled us to perform their meta-analysis in order to understand the precise association between this polymorphism and cancer risk. For meta-analysis a literature search following stringent criteria, as stated in the methodology section, was adopted. A total of eight studies regarding *LMP7* –145 (C > A) gene polymorphism and cancer risk were found eligible for inclusion in this meta-analysis. All the retrieved articles were examined carefully by reading their titles and abstracts, and the full texts of the potentially germane publications were further reviewed for their appropriateness for this meta-analysis. Published studies either showing *LMP* polymorphism to predict survival in cancer patients or considering *LMP* variants as an indicators for response-to-therapy were disqualified straightaway. Likewise, studies related to the investigation of the levels of LMP mRNA or protein expression or pertinent review articles were also disqualified. In this meta-analysis, only case-control or cohort design clinical studies having frequency of all the three genotypes were included. In addition to the online database search, the references listed in the primary retrieved articles were also examined for other relevant potential articles. The major characteristics of all the eight studies included in this meta-analysis, i.e., distribution of genotypes, minor allele frequency (MAF) in controls and cases have been given in Table 1 and Table 2. All the eight studies included in this meta-analysis were appraised for the quality score according to the NOS analysis, and almost all the studies (< 80%) scored 5 stars or more, and suggested a moderate to good quality (Table 3). The needful information related to the selection and inclusion of the studies for this meta-analysis has been given in Figure 1 (PRISMA 2009 Flow Diagram).

**Table 1:** Main characteristics of all the 8 studies included in the present meta-analysis

First author, (Year) Ref. No.	Country	Ethnicity	Type of cancer	Type of study	Controls	Cases	Methods	Association
Ma et al. (2015) [20]	China	Asian	Gastric	HB	502	502	PCR-RFLP	Yes
Mehta et al. (2015) [21]	Indonesia	Asian	Cervical	PB	173	201	TaqMan SNP Genotyping Assay	No
Song et al. (2014) [22]	China	Asian	Ovarian	HB	338	235	PCR-RFLP	Yes
Ozbas et al. (2013) [23]	Turkey	Asian	Hematological malignancy	HB	130	132	PCR-RFLP	Yes
Fellerhoff et al. (2011) [24]	Germany	Caucasian	Colon	HB	165	174	ARMS-PCR	Yes
Deshpande et al. (2008) [25]	USA	Caucasian	Cervical	PB	224	134	Sequencing	No
Mehta et al. (2007) [26]	Netherlands	Caucasian	Cervical	HB	124	127	TaqMan SNP	Yes
Cao et al. (2005) [27]	China	Asian	Esophageal	HB	357	265	Sequencing	Yes

HB: Hospital base; PB: Population base.

**Table 2:** Genotypic distribution of LMP7 -145 C > A gene polymorphism included in this meta-analysis

First authors (year)	Controls				Cases				HWE
	Genotype			Minor allele	Genotype			Minor allele	
	CC	CA	AA	MAF	CC	CA	AA	MAF	*p*-value
Ma et al. (2015)	349	141	12	0.164	310	169	23	0.214	0.612
Mehta et al. (2015)	141	22	1	0.073	173	18	1	0.052	0.888
Song et al. (2014)	249	76	13	0.150	120	86	29	0.306	0.024
Ozbas et al. (2013)	112	17	1	0.073	111	18	3	0.090	0.692
Fellerhoff et al. (2011)	145	20	0	0.060	97	70	7	0.241	0.407
Deshpande et al. (2008)	182	41	1	0.095	106	27	1	0.108	0.412
Mehta et al. (2007)	78	43	3	0.197	96	31	0	0.122	0.297
Cao et al. (2005)	210	130	17	0.229	130	114	21	0.294	0.583

MAF: Minor allele frequency, HWE: Hardy weinberg equilibrium.

**Table 3:** Quality assessment for all the studies included in this meta-analysis according to the Newcastle-Ottawa scale

First author (year)	Quality indicators		
	Selection	Comparability	Exposure
Ma et al. (2015)	****	*	**
Mehta et al. (2015)	**	*	**
Song et al. (2014)	***	*	**
Ozbas et al. (2013)	***	*	**
Fellerhoff et al. (2011)	***	*	**
Deshpande et al. (2008)	****	*	**
Mehta et al. (2007)	***	*	**
Cao et al. (2005)	****	*	**

### Detection of publication bias

The Begg’s funnel plot and Egger’s test were performed to appraise the publication bias among the clinical studies of *LMP7* –145 (C > A) gene polymorphism included in this meta-analysis. No evidence of publication bias was observed by the appearance of the shape of funnel plots and the results of Egger’s test in all the five genetic models of *LMP7* –145 (C > A) (Table 4, Supplementary Information: Supplementary Figure 1) gene polymorphism.

**Table 4:** Statistics to test publication bias and heterogeneity of LMP7 -145 C > A gene polymorphism

Comparisons	Egger’s regression analysis			Heterogeneity analysis			Model used for the meta-analysis
	Intercept	95% Confidence Interval	*p*-value	*Q*-value	P_heterogeneity_	I^2^ (%)	
**Overall risk**							
A vs C	−1.27	−8.25 to 5.70	0.67	55.96	0.01	87.49	Random
AA vs CC	−0.47	−2.48 to 1.52	0.57	10.64	0.15	34.26	Fixed
AC vs CC	−1.16	−8.29 to 5.95	0.70	43.99	0.01	84.09	Random
AA + AC vs CC	−1.42	−9.07 to 6.22	0.66	53.57	0.01	86.93	Random
AA vs AC + CC	−0.35	−2.09 to 1.39	0.63	8.090	0.32	13.51	Fixed
**Asian risk**							
A vs C	−1.98	−11.03 to 7.06	0.53	18.83	0.01	78.76	Random
AA vs CC	−0.55	−4.18 to 3.07	0.66	4.210	0.37	5.020	Fixed
AC vs CC	−2.04	−9.80 to 5.71	0.46	12.54	0.01	68.11	Random
AA + AC vs CC	−2.22	−11.34 to 6.90	0.49	16.86	0.01	76.22	Random
AA vs AC + CC	−0.33	−3.42 to 2.75	0.75	2.980	0.56	0.01	Fixed
**Caucasian risk**							
A vs C	164.81	143.45 to 186.16	0.00	36.90	0.01	94.58	Random
AA vs CC	−26.74	−590.48 to 536.99	0.65	6.200	0.04	67.77	Random
AC vs CC	170.25	−2043.43 to 2383.94	0.50	31.42	0.01	93.63	Random
AA+AC vs CC	154.88	−2017.17 to 2326.93	0.53	36.67	0.01	94.54	Random
AA vs AC+CC	−25.34	−525.35 to 474.67	0.63	4.940	0.08	59.58	Fixed

### Evaluation of heterogeneity

*Q*-test and I^2^ statistics were employed to test heterogeneity among the selected genetic association studies of *LMP7*–145 (C > A) gene polymorphism and cancer susceptibility. Significant heterogeneity was observed in three models of *LMP7*–145 (C > A). Thus, random effects model was used to synthesize the data (Table 4).

### Association of LMP7–145 C > A polymorphism and overall cancer susceptibility

**Figure 2:**
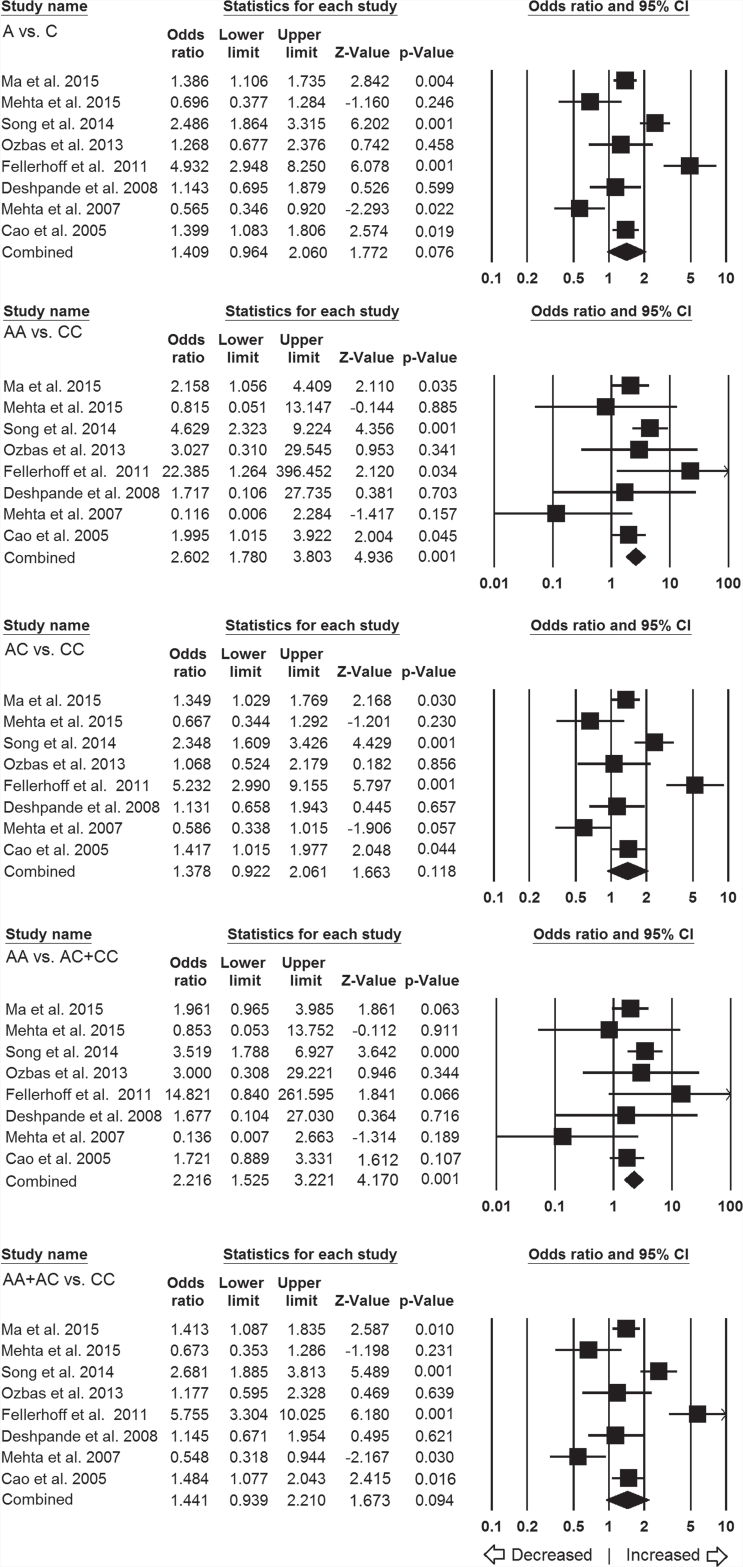
Association of LMP7–145 C > A polymorphism and cancer susceptibility in overall population Forest plot of ORs with 95% CI of cancer risk associated with the *LMP7* –145 C > A gene polymorphism for overall population. Note: Black square represents the value of OR and the size of the square indicates the inverse proportion relative to its variance. Horizontal line is the 95% CI of OR.

A total of eight studies were qualified for inclusion in this analysis to assess the overall association between *LMP7*−145 (C > A) polymorphism and cancer risk. Pooled analysis of total 1770 different cancer cases and 2013 controls demonstrated that homozygous AA genotype was significantly associated with 2.6 folds increased risk of overall cancer as compared to the CC genotype (AA vs CC: *p* = 0.001; OR = 2.602, 95% CI = 1.780 to 3.803). The recessive genetic model also demonstrated OR of 2.21 (AA vs AC+CC: *p* = 0.001; OR = 2.216, 95% CI = 1.525 to 3.221), indicating that individuals with AA genotype had almost 2 folds higher risk of cancer than those with the AC and CC genotypes. However, other genetic models, allelic (A vs C: *p* = 0.076; OR = 1.409, 95% CI = 0.964 to 2.060), heterozygous (AC vs CC: *p* = 0.118; OR = 1.378, 95% CI = 0.922 to 2.061), dominant model (AA+AC vs CC: *p* = 0.094; OR = 1.441, 95% CI = 0.939 to 2.210) did not show any association (Figure 2).

**Figure 3:**
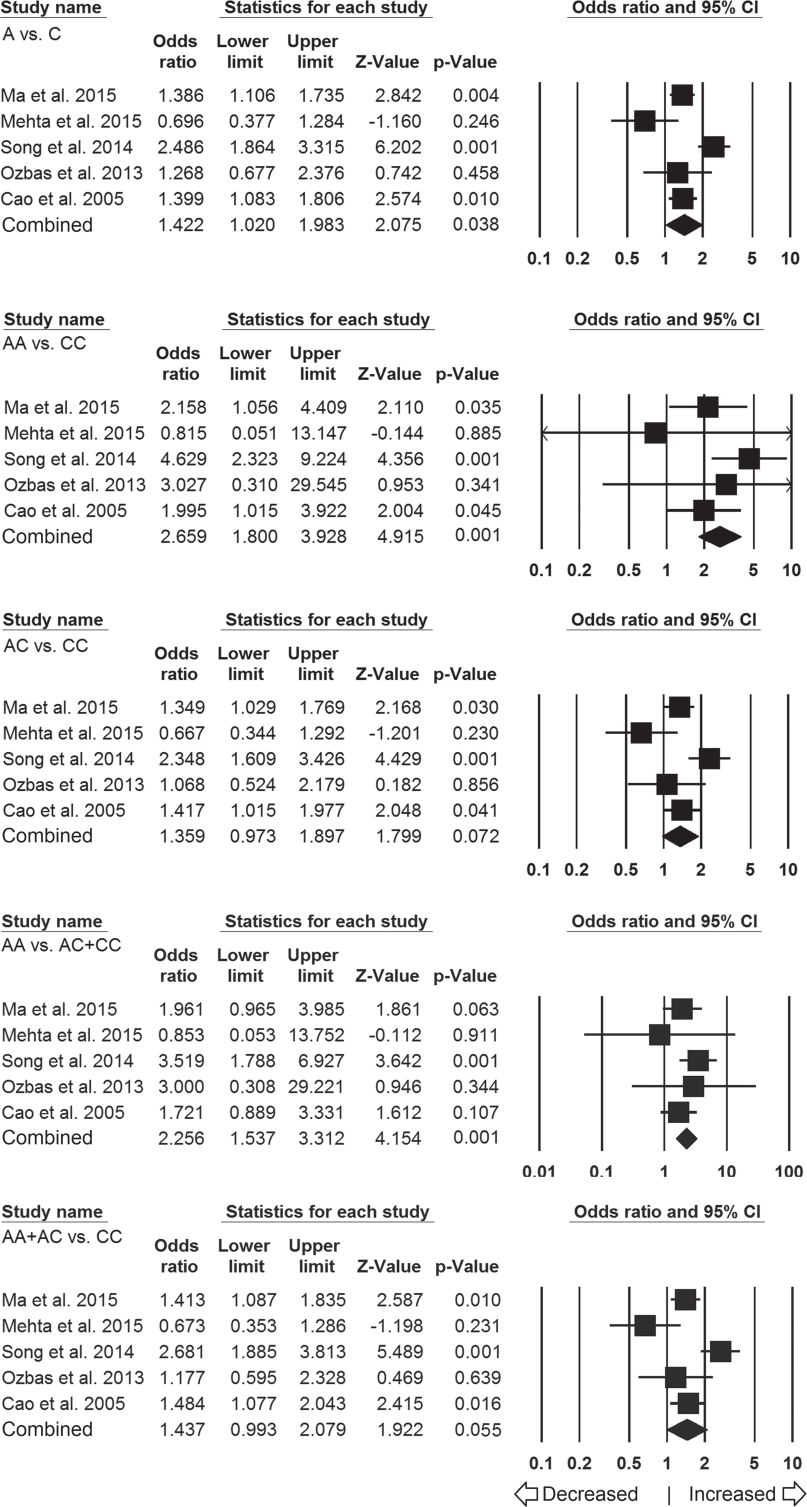
Association of LMP7–145 C > A polymorphism and cancer susceptibility in Asian subpopulation Forest plot of ORs with 95% CI of cancer risk associated with the *LMP7*–145 C > A gene polymorphism for Asian subgroup population. Note: Black square represents the value of OR and the size of the square indicates the inverse proportion relative to its variance. Horizontal line is the 95% CI of OR.

In ethnicity-wise subgroup analysis, a total of five studies were found qualified for Asian population and comprised of 1500 controls and 1335 different cancer cases. Heterogeneity was observed in three genetic models, so random effects model was applied to generate ORs and 95% CIs (Table 4). The results of Asian subgroup analysis suggested 2.2 folds statistically significant increased risk of cancer with homozygous AA (AA vs CC: *p* = 0.001; OR = 2.659, 95% CI = 1.800 to 3.928) and recessive genetic models (AA vs AC+CC: *p* = 0.001; OR = 2.256, 95% CI = 1.537 to 3.312) in comparison with wild type CC genotype. In addition, variant allele also revealed 1.4 folds increased risk of developing cancer (A vs C: *p* = 0.038; OR = 1.422, 95% CI = 1.020 to 1.983). Marginally significant risk was also observed for the dominant (AA+AC vs CC: *p* = 0.055; OR = 1.437, 95% CI = 0.993 to 2.079) genetic model, whereas, heterozygous (AC vs CC: *p* = 0.072; OR = 1.359, 95% CI = 0.973 to 1.897) model failed to show any risk (Figure 3).

**Figure 4:**
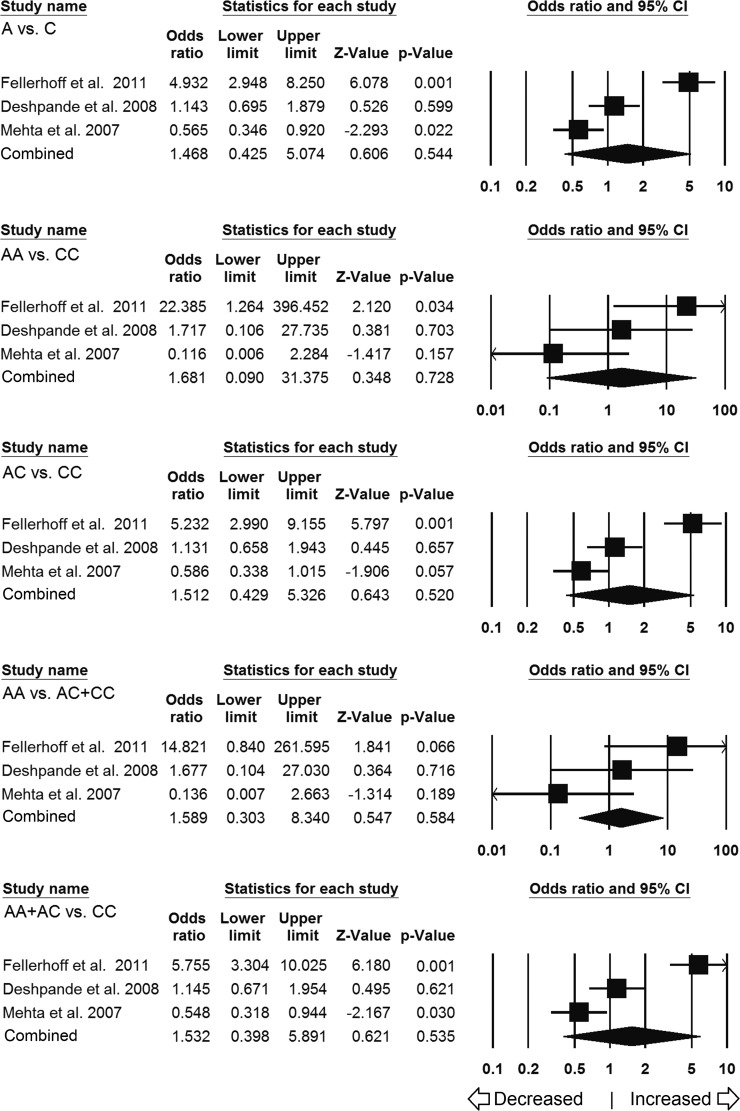
Association of LMP7 –145 C > A polymorphism and cancer susceptibility in caucasian subpopulation Forest plot of ORs with 95% CI of cancer risk associated with the *LMP7* –145 C > A gene polymorphism for Caucasian subgroup population. Note: Black square represents the value of OR and the size of the square indicates the inverse proportion relative to its variance. Horizontal line is the 95% CI of OR.

For ethnicity-wise subgroup analysis of Caucasian population, only three studies (including 513 controls and 435 different cancer cases) were qualified and included for this analysis. Heterogeneity was observed in four genetic models; hence, random effects model was applied to generate pooled ORs and corresponding 95% CI values (Table 4). No obvious relevance of *LMP7–*145 (C > A) variant with cancer susceptibility was observed in all the genetic models (Figure 4), i.e., allelic (A vs C: *p* = 0.544; OR = 1.468, 95% CI = 0.425 to 5.074), homozygous (AA vs CC: *p* = 0.728; OR = 1.681, 95% CI = 0.090 to 31.375), heterozygous (AC vs CC: *p* = 0.520; OR = 1.512, 95% CI = 0.429 to 5.326), dominant model (AA+AC vs CC: *p* = 0.535; OR = 1.532, 95% CI = 0.398 to 5.891) and recessive (AA vs AC+CC: *p* = 0.584; OR = 1.589, 95% CI = 0.303 to 8.340).

### Sensitivity analysis for LMP7–145 C > A gene polymorphism and cancer susceptibility

To evaluate the impact of individual study on the risk of overall cancer, we performed leave-one-out sensitivity analysis and calculated the pooled ORs. The ORs recomputed after excluding each single study did not show any difference from their primary values, and assured the stability of the overall results (Supplementary Figure 2). Furthermore, the estimated pooled ORs in Asian (Supplementary Figure 3) and Caucasian (Supplementary Figure 4) subgroup populations did not change, which showed that the results of subgroup analysis were also stable.

### Trial sequential analysis of LMP7–145 C > A gene polymorphism and cancer risk

**Figure 5:**
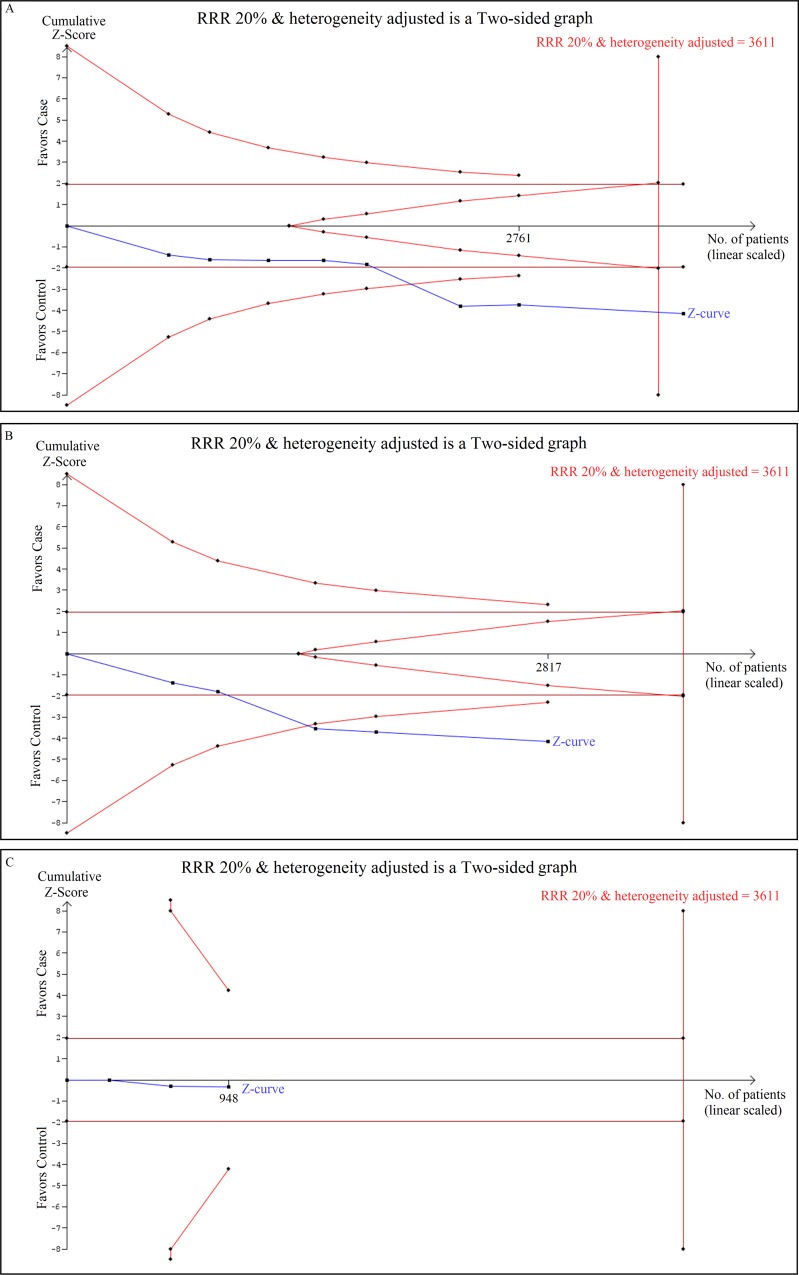
Trial sequential analysis of LMP7–145 C > A gene polymorphism and cancer risk Trial sequence analysis of all studies on *LMP7* –145 C > A gene polymorphism based on recessive genetic model. (**A**) In overall cancer risk (**B**) Cancer risk among Asian population (**C**) Cancer risk among Caucasian population.

The results of TSA were consistent with the conventional meta-analysis in the case of *LMP7* –145 C > A gene polymorphism as the cumulative Z curve crossed with TSA monitoring boundary confirming that no further relevant trials are necessary. TSA analysis using recessive genetic (AA vs AC+CC) model, for e.g., showed that meta-analysis had enough number of studies (required sample size = 3611, achieved 80% power) for a significant conclusion, as it crossed the O’Breien-Fleming boundary (Figure 5A). Similarly, the cumulative Z curve crossed with TSA monitoring boundary before reaching the required information size in Asian subjects, confirming that *LMP7* –145 C > A polymorphism is associated with increased cancer risk and further relevant trials are unnecessary (Figure 5B). Whereas, for Caucasian population, the cumulative Z curve failed to cross the trial monitoring boundary before reaching the required power and indicated that the cumulative evidence is insufficient and further trials are necessary (Figure 5C).

## DISCUSSION

Despite innumerable cutting edge improvements in diagnostic and therapeutic techniques, cancer remains the most lethal disease all over the world. Genetic variants that are important factor in immunogenesis are gradually being recognized as clues to individual’s susceptibility for cancer, exploring why individuals with shared environmental exposures do not always share cancer related morbidity and mortality.

The LMP7 protein is considered as catalytic subunit of the immunoproteasome, and can be induced with interferon-γ ensuing distinct subunit composition and altered catalytic characteristics [30]. As being important subunits of the immunoproteasome, the proteins of *LMP7* have significant roles in antigen presentation and therefore they have played a suspected factor for a large variety of autoimmune diseases including cancer. Endogenous antigen presentation is a pivotal mechanism for the recognition of virally infected cells, maintenance of self-tolerance, and the surveillance of newly arising tumors by the immune system [31]. It is commonly anticipated that the functions of antigen processing and transport pathway might be impacted by the structural differences encoded by TAP (transporter associated with antigen processing) and LMP alleles, and immunoproteasome advances the quality and quantity of the generated class-I ligands. In the human host’s protective immunity, LMP/TAP system may perhaps detects tumor antigens and plays a key role in the immune surveillance via MHC-I molecule and CTL [32]. The genes that encode above said proteins are polymorphic in nature, hence it is possible that a particular genotype/allele of *LMP7* –145 C > A polymorphism affect the functional alternations that may lead to the production of an insufficient peptide level, which may allow cancer cell to escape immune processing and lead to cancer development. Earlier studies already proved that individual studies with a low sample size may have not sufficient statistical power to identify a small risk factor. Hence, it is quite rational to appraise the precise association of *LMP7* –145 C > A gene variant to understand the contribution in overall cancer risk.

To the best of our knowledge, this is the very first meta-analysis evaluating the association between *LMP7* –145 C > A gene polymorphism and overall cancer risk for obtaining a precise conclusion. In the present study, we pooled all eight qualified case-control studies retrieved from different online web-databases that supported the key role of *LMP7* −145 C > A polymorphism in overall cancer risk. More specifically, the AA genotype of *LMP7* −145 C > A gene polymorphism had significantly greater risk of developing cancer in comparison with the wild type CC genotype. Subgroup analysis by ethnicity also demonstrated a similar trend of an increased risk of overall cancer in Asian population. Whereas, no association of *LMP7* −145 C > A gene polymorphism with cancer risk was observed in Caucasian population.

In addition, the potential association of *LMP7* −145 C > A polymorphism with overall cancer risk was confirmed by the Trial Sequential analysis, which further strengthen the conclusion that *LMP7* −145 C > A polymorphism confers an increased risk of cancer. Overall, the pooled analysis suggested that genetic polymorphisms in the *LMP7* gene may affect its expression level and play a crucial role in the failure of immunosurveillance and contribute to cancer progression. This is in line with previously published reports where they observed low protein expression and protein down regulation of *LMP7* in several malignancies [31, 33, 34].

Although, the etiology of cancer is polygenic in nature and the roles of *LMP7* polymorphism in the risk of developing cancer are quite diverse. Hence, a single genetic polymorphism is usually inadequate to clarify the susceptibility of this complex disease possibly because of significant heterogeneity in the clinical features and acquired genetic alterations.

This meta-analysis has certain limitations that must be discussed for better understanding and for the future studies, for e.g., first, heterogeneity was present in some genetic models and thus the random-effects model was applied to obtain the broader CI values. Second, due to non-availability of the original data for each included study, we failed to adjust the pooled ORs with respect to subjects’ age, sex, and environmental factors.

Despite above mentioned minor limitations, the current study has some major advantages, like, in order to check the credibility of the findings, we performed the publication bias analysis and the generated funnel plots suggested no obvious publication bias among the selected studies. This suggests that the results of the present meta-analysis are statically robust. Sensitivity analysis also indicated that no single study yield obvious impact on the pooled ORs and corresponding CIs. The credibility of all the eight included studies in terms of their quality was also checked using NOS scale. Additionally, in order to reconfirm the results of meta-analysis, TSA was performed and the results of TSA suggested that the conclusions were robust. Moreover, explicit criteria for the study examination and inclusion, strict data extraction, and exhaustive pooled analysis were applied to draw satisfactory and reliable conclusion.

In summary, the overall results of the present meta-analysis suggested that individuals with *LMP7*−145 C > A genetic polymorphism have increased cancer risk. These results will improve our understanding of the role of *LMP7*−145 C > A genetic polymorphism and assist in identification of at-risk individuals. Furthermore, this increasing knowledge could imply for other aspects of cancer management, including prevention, screening, and therapeutic treatment.

Also, future larger studies comprising other relevant factors along with integrative network modules analysis are warranted to clarify the potential role of *LMP7* genetic variant in cancer risk.

## MATERIALS AND METHODS

### Identification and eligibility of the relevant studies

A systematic search was performed using PubMed (Medline), Google Scholar and EMBASE web-databases to retrieve compatible and peer reviewed research articles for this meta-analysis. Last search was updated on January 2017 with the combination of following keywords: ‘low molecular weight polypeptide 7 OR *LMP7* OR *PSMB8* gene (polymorphism OR mutation OR variant) AND cancer susceptibility OR risk. The search was limited on published studies that had been conducted in human subjects only. All the retrieve articles were examined by reading their titles and abstracts, and all the published studies matching with the above said eligibility criteria were selected for this meta-analysis. We also did manual search of the reference list from the retrieve articles for other eligible pertinent studies.

### Inclusion and exclusion criteria

All the published articles included in the current meta-analysis had to meet all the given criteria: a) it must evaluated the association between *LMP7* −145 C > A polymorphisms and cancer risk, b) used a case-control design, c) recruited histologically confirmed cancer patients and healthy controls, d) have available genotype frequency in cases and controls, e) and must be published in the English language. In addition to above, when the same patient/subject populations appeared in several publications, only the most recent one or the complete study was included in this meta-analysis.

The major reasons for study exclusion for this pooled analysis were overlapping of the data, case-only studies, review articles, and lack of genotype frequencies or number not reported. The information related to the selection (inclusion/ exclusion) of the studies is appended as Figure 1 in the form of PRISMA 2009 Flow Diagram.

### Data extraction

For each retrieved article, the methodological quality assessment and data extraction were independently summarized in duplicate by two independent investigators (RKM & SAD) using a standard procedure. Data-collection form was used to ensure the accuracy of the collected data by strictly following the inclusion/ exclusion criteria as stated above. The major characteristic summarized from the retrieved articles included, the name of the first author, year of publication, the country of origin, the number of cases and controls, type of cancer, type of study, association/no-association status, methods of genotyping and genotype frequencies for the cases and controls. The cases related with disagreement on any item of the data from the collected studies were fully debated with the investigators in the presence of adjudicator (SH) to attain a final consensus.

### Quality assessment by Newcastle-Ottawa scale (NOS) criteria

The methodological quality assessment of the included studies was also done separately by two independent investigators (RKM & SAD) using the NOS criteria. The NOS criteria majorly included three aspects: (1) subject selection: 0–4 points; (2) comparability of subjects: 0−2 points; (3) clinical outcomes: 0−3 points. The studies that were awarded 5 stars or more can be considered as of moderate to high quality [35]. The assessment was performed independently by two investigators (RKM & SAD, as stated above) and the inconformity was resolved by a discussion or consultation if necessary (with the help of adjudicator SH).

### Statistical analysis

The potential association between studied *LMP7* single nucleotide polymorphism and cancer susceptibility was assessed by computing crude Odds Ratios (ORs) and the corresponding 95% CI. The pooled ORs were estimated for the allele contrast, log-additive, dominant, and recessive models [36]. Heterogeneity assumptions between the studies across the eligible comparisons were performed by the chi-square-based *Q*-test [37]. Heterogeneity was considered significant when *p*-value < 0.05 to avoid underestimation of the presence of heterogeneity. A fixed effect model (if *p* > 0.05) [38] or a random effect model (if *p* < 0.05) [39] was used for pooling the results. Moreover, I^2^ statistics was also applied to efficiently test the heterogeneity among the selected studies [40]. Hardy-Weinberg equilibrium (HWE) in the controls was estimated via Chi-square test. Likewise, the Funnel plot asymmetry was estimated by Egger’s linear regression test, which is a type of linear regression approach to measure the funnel plot asymmetry on the natural logarithm scale of the ORs. The significance of the intercept was determined by the *t*-test (*p*-value <0.05 was considered as representation of statistically significant publication bias) [41]. All the statistical calculations were done by using Comprehensive Meta-Analysis (CMA) Version2 software program (Biostat, NJ, USA). All the *p*-values were two sided, and the statistical significance was considered as *p*-value less than 0.05 for this meta-analysis.

### Trial sequential analysis (TSA)

According to Cochrane handbook, meta-analyses are considered to be the best if all the eligible trials are included in the analysis. However, it may not be sufficient evidence. Occasionally, the meta-analysis may lead to systematic errors (bias) or random errors (play of chance). In order to minimize errors, a novel statistical analysis based software program, TSA (Trial sequential analysis tool from Copenhagen Trial Unit, Center for Clinical Intervention Research, Denmark) has been introduced, which estimates the required information size, and an adjusted threshold for the statistical significance, and estimates the power of the current conclusion [42–44]. If the TSA monitoring boundary crossed with Z curve before the required information size is reached, robust evidence might have been confirmed, and further trails are unnecessary. In contrast, if the Z curve does not cross monitoring boundaries and the required information size has not been reached, it is necessary to continue doing trials. In the present investigation, Trial Sequential Analysis (Version 0.9, http://www.ctu.dk/tsa/) software program was used for the TSA analysis.

## SUPPLEMENTARY MATERIALS FIGURES


