# Supplementary material for: Impact of LMP7 (rs2071543) gene polymorphism in increasing cancer risk: evidence from a meta-analysis and trial sequential analysis
Source: Oncotarget. 2017 Dec 21;9(5):6572–85. doi: 10.18632/oncotarget.23547 (PMC5814233; doi:10.18632/oncotarget.23547)
Supplement: Supplementary file 1 [file oncotarget-09-6572-s001.pdf]

Impact of *LMP7* (rs2071543) gene polymorphism in increasing cancer risk: evidence from a meta-analysis and trial sequential analysis

SUPPLEMENTARY MATERIALS

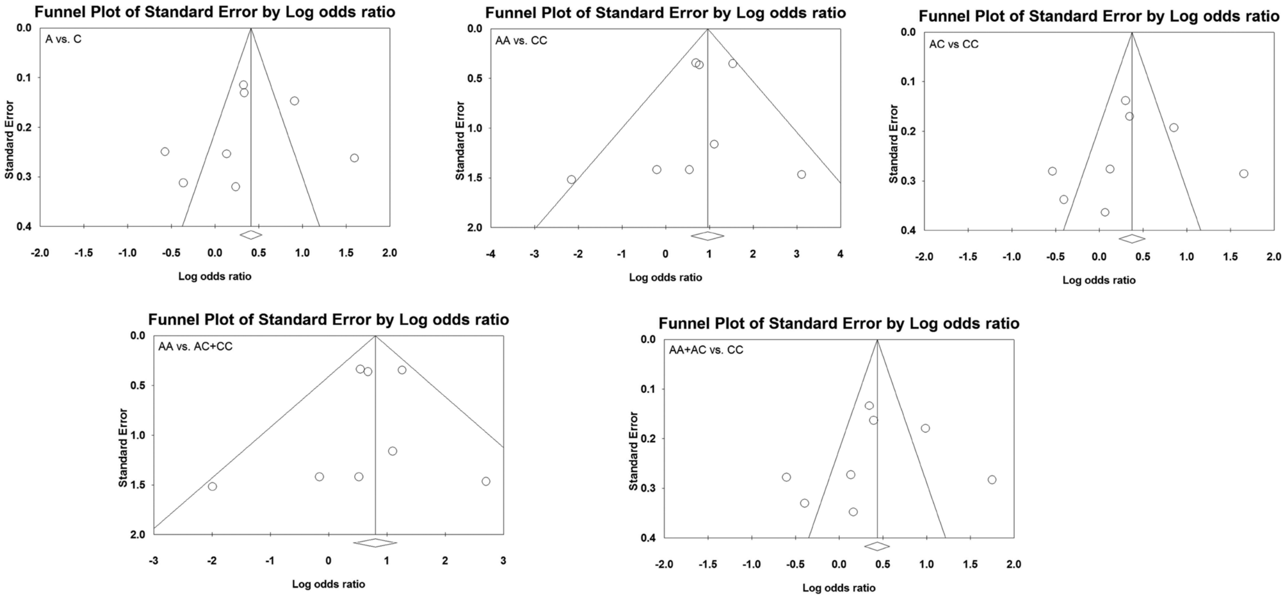

Supplementary Figure 1: Assessment of publication bias shown with Funnel plots in studies assaying odds of cancer associated with the LMP7 -145 C > A gene polymorphism for overall analysis (Odds ratio against standard error in different genetic models).

Study name<br>A vs. C	Point	Lower limit	Upper limit	Z-Value	p-Value
Ma et al. 2015	1.405	0.865	2.283	1.374	0.169
Mehta et al. 2015	1.536	1.037	2.276	2.141	0.032
Song et al. 2014	1.286	0.861	1.922	1.228	0.220
Ozbas et al. 2013	1.426	0.942	2.159	1.678	0.093
Fellerhoff et al. 2011	1.208	0.864	1.689	1.104	0.270
Deshpande et al. 2008	1.449	0.952	2.205	1.728	0.084
Mehta et al. 2007	1.604	1.119	2.299	2.571	0.010
Cao et al. 2005	1.404	0.874	2.255	1.404	0.169
	1.409	0.964	2.060	1.772	0.076

Statistics with study removed

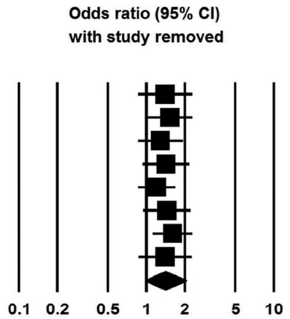

Study name<br>AA vs. CC	Point	Lower limit	Upper limit	Z-Value	p-Value
Ma et al. 2015	2.800	1.789	4.384	4.503	0.001
Mehta et al. 2015	2.660	1.813	3.902	5.002	0.001
Song et al. 2014	2.025	1.285	3.191	3.040	0.002
Ozbas et al. 2013	2.590	1.763	3.807	4.845	0.001
Fellerhoff et al. 2011	2.504	1.707	3.673	4.697	0.001
Deshpande et al. 2008	2.622	1.787	3.847	4.930	0.001
Mehta et al. 2007	2.739	1.868	4.016	5.159	0.001
Cao et al. 2005	2.940	1.858	4.653	4.606	0.001
	2.602	1.780	3.803	4.936	0.001

Statistics with study removed

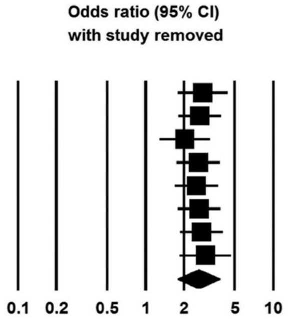

Study name<br>AC vs. CC	Point	Lower limit	Upper limit	Z-Value	p-Value
Ma et al. 2015	1.374	0.825	2.291	1.220	0.222
Mehta et al. 2015	1.508	0.994	2.287	1.932	0.053
Song et al. 2014	1.266	0.816	1.965	1.051	0.293
Ozbas et al. 2013	1.419	0.915	2.199	1.564	0.118
Fellerhoff et al. 2011	1.176	0.849	1.629	0.973	0.331
Deshpande et al. 2008	1.414	0.901	2.218	1.507	0.132
Mehta et al. 2007	1.554	1.049	2.303	2.198	0.028
Cao et al. 2005	1.365	0.834	2.234	1.239	0.215
	1.378	0.922	2.061	1.663	0.118

Statistics with study removed

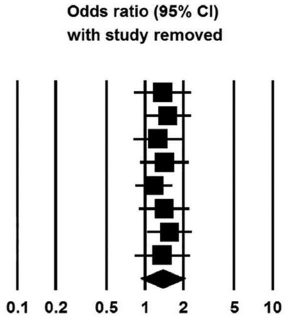

Study name<br>AA vs. AC+CC	Point	Lower limit	Upper limit	Z-Value	p-Value
Ma et al. 2015	2.323	1.496	3.608	3.753	0.001
Mehta et al. 2015	2.255	1.546	3.290	4.224	0.001
Song et al. 2014	1.809	1.155	2.833	2.589	0.010
Ozbas et al. 2013	2.198	1.504	3.211	4.070	0.001
Fellerhoff et al. 2011	2.145	1.471	3.127	3.964	0.001
Deshpande et al. 2008	2.227	1.527	3.249	4.159	0.001
Mehta et al. 2007	2.318	1.590	3.379	4.370	0.001
Cao et al. 2005	2.497	1.586	3.931	3.952	0.001
	2.216	1.525	3.221	4.170	0.001

Statistics with study removed

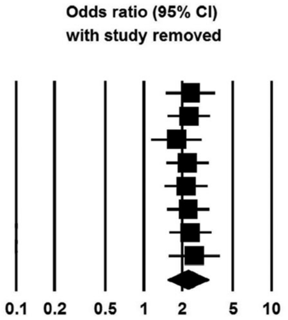

Study name<br>AA+AC vs. CC	Point	Lower limit	Upper limit	Z-Value	p-Value
Ma et al. 2015	1.437	0.834	2.476	1.306	0.191
Mehta et al. 2015	1.586	1.018	2.472	2.038	0.042
Song et al. 2014	1.307	0.826	2.066	1.143	0.253
Ozbas et al. 2013	1.475	0.924	2.356	1.629	0.103
Fellerhoff et al. 2011	1.213	0.842	1.748	1.037	0.300
Deshpande et al. 2008	1.485	0.922	2.393	1.626	0.104
Mehta et al. 2007	1.651	1.094	2.490	2.389	0.017
Cao et al. 2005	1.428	0.845	2.413	1.329	0.184
	1.441	0.939	2.210	1.673	0.094

Statistics with study removed

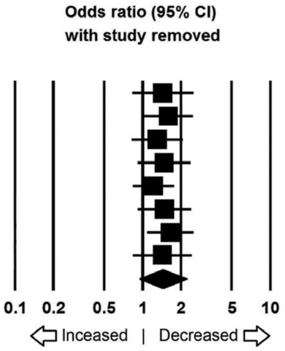

Supplementary Figure 2: Sensitivity analysis ofLMP7 -145 C > A gene polymorphism with overall cancer risk to evaluate the influence of each individual study on the pooled OR by deleting one single study each time for overall analysis (for all the genetic models). Black square represents the value ofOR and the size of the square indicates the inverse proportion relative to its variance. Horizontal line is the 95% CI of OR.

A vs. C

Study name	Point	Lower limit	Upper limit	Z-Value	p-Value
Ma et al. 2015	1.396	0.865	2.255	1.365	0.172
Mehta et al. 2015	1.608	1.180	2.190	3.011	0.003
Song et al. 2014	1.278	1.026	1.591	2.191	0.028
Ozbas et al. 2013	1.442	0.988	2.104	1.897	0.058
Cao et al. 2005	1.395	0.872	2.231	1.389	0.165
	1.422	1.020	1.983	2.075	0.038

AA vs. CC

Study name	Point	Lower limit	Upper limit	Z-Value	p-Value
Ma et al. 2015	2.906	1.824	4.628	4.491	0.001
Mehta et al. 2015	2.723	1.836	4.038	4.984	0.001
Song et al. 2014	2.049	1.277	3.288	2.972	0.003
Ozbas et al. 2013	2.649	1.783	3.935	4.823	0.001
Cao et al. 2005	3.069	1.904	4.948	4.602	0.001
	2.659	1.800	3.928	4.915	0.001

AC vs. CC

Study name	Point	Lower limit	Upper limit	Z-Value	p-Value
Ma et al. 2015	1.320	0.810	2.150	1.114	0.265
Mehta et al. 2015	1.536	1.148	2.055	2.888	0.004
Song et al. 2014	1.220	0.940	1.583	1.496	0.135
Ozbas et al. 2013	1.402	0.962	2.043	1.760	0.078
Cao et al. 2005	1.306	0.816	2.090	1.112	0.266
	1.359	0.973	1.897	1.799	0.072

AA vs. AC+CC

Study name	Point	Lower limit	Upper limit	Z-Value	p-Value
Ma et al. 2015	2.391	1.515	3.774	3.742	0.001
Mehta et al. 2015	2.299	1.560	3.387	4.210	0.001
Song et al. 2014	1.827	1.147	2.912	2.535	0.011
Ozbas et al. 2013	2.237	1.516	3.303	4.052	0.001
Cao et al. 2005	2.590	1.616	4.152	3.953	0.001
	2.256	1.537	3.312	4.154	0.001

AA+AC vs. CC

Study name	Point	Lower limit	Upper limit	Z-Value	p-Value
Ma et al. 2015	1.405	0.824	2.396	1.249	0.212
Mehta et al. 2015	1.654	1.183	2.311	2.946	0.003
Song et al. 2014	1.268	0.968	1.661	1.727	0.084
Ozbas et al. 2013	1.479	0.971	2.252	1.824	0.068
Cao et al. 2005	1.389	0.828	2.330	1.246	0.213
	1.437	0.993	2.079	1.922	0.055

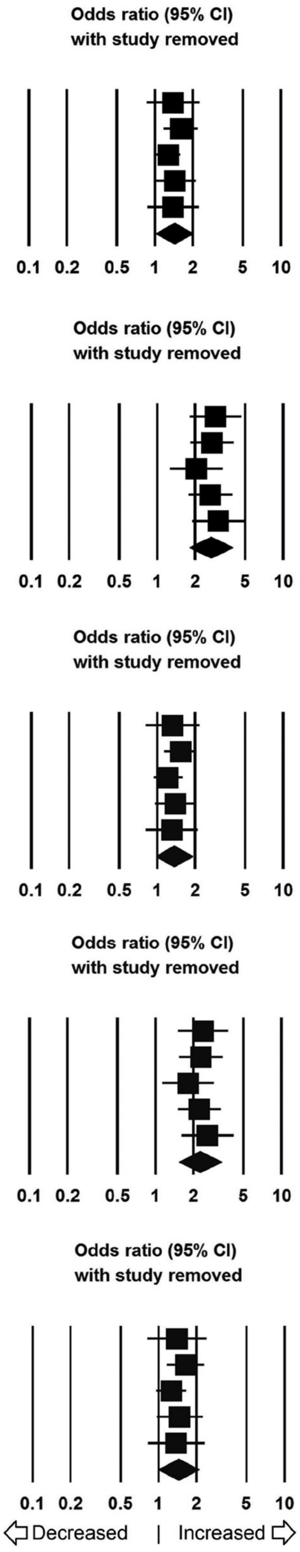

Supplementary Figure 3: Sensitivity analysis of LMP7 -145 C > A gene polymorphism with cancer risk among Asian population to evaluate the influence of each individual study on the pooled OR by deleting one single study each time for overall analysis (for all the genetic models). Black square represents the value of OR and the size of the square indicates the inverse proportion relative to its variance. Horizontal line is the 95% CI of OR.

Study name A vs. C — **Statistics with study removed**

Study name	Point	Lower limit	Upper limit	Z-Value	p-Value
Fellerhoff et al. 2011	0.802	0.402	1.601	-0.626	0.531
Deshpande et al. 2008	1.666	0.199	13.936	0.471	0.638
Mehta et al. 2007	2.370	0.566	9.934	1.181	0.238
	1.468	0.425	5.074	0.606	0.544

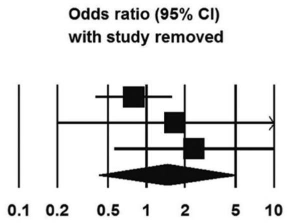

Study name AA vs. CC — **Statistics with study removed**

Study name	Point	Lower limit	Upper limit	Z-Value	p-Value
Fellerhoff et al. 2011	0.472	0.034	6.590	-0.558	0.577
Deshpande et al. 2008	1.637	0.009	283.836	0.187	0.851
Mehta et al. 2007	6.038	0.488	74.735	1.401	0.161
	1.681	0.090	31.375	0.348	0.728

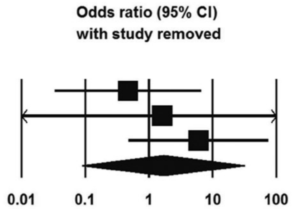

Study name AC vs. CC — **Statistics with study removed**

Study name	Point	Lower limit	Upper limit	Z-Value	p-Value
Fellerhoff et al. 2011	0.815	0.428	1.553	-0.621	0.535
Deshpande et al. 2008	1.750	0.205	14.957	0.511	0.609
Mehta et al. 2007	2.428	0.541	10.896	1.158	0.247
	1.512	0.429	5.326	0.643	0.520

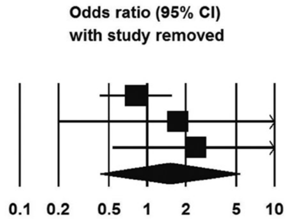

Study name AA vs AC+CC — **Statistics with study removed**

Study name	Point	Lower limit	Upper limit	Z-Value	p-Value
Fellerhoff et al. 2011	0.520	0.068	3.961	-0.631	0.528
Deshpande et al. 2008	1.543	0.196	12.168	0.411	0.681
Mehta et al. 2007	4.814	0.653	35.467	1.542	0.123
	1.589	0.303	8.340	0.547	0.584

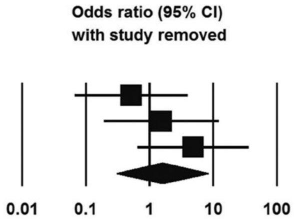

Study name AA+AC vs. CC — **Statistics with study removed**

Study name	Point	Lower limit	Upper limit	Z-Value	p-Value
Fellerhoff et al. 2011	0.793	0.385	1.634	-0.628	0.530
Deshpande et al. 2008	1.774	0.177	17.789	0.487	0.626
Mehta et al. 2007	2.562	0.526	12.472	1.165	0.244
	1.532	0.398	5.891	0.621	0.535

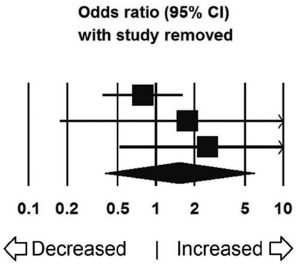

Supplementary Figure 4: Sensitivity analysis ofLMP7 -145 C > A gene polymorphism with cancer risk among Caucasian population to evaluate the influence of each individual study on the pooled OR by deleting one single study each time for overall analysis (for all the genetic models). Black square represents the value ofOR and the size of the squareindicates the inverse proportion relative to its variance. Horizontal line is the 95% CI of OR.
